# Unusual presentation of cactus spines in the flank of an elderly man: a case report

**DOI:** 10.1186/1752-1947-4-152

**Published:** 2010-05-25

**Authors:** Andrea Suárez, Scott Freeman, Lauren Puls, Robert Dellavalle

**Affiliations:** 1 University of Colorado Hospital, Dermatology Clinic, Mail Stop F703, PO Box 6510, Aurora, CO 80045-0510, USA; 2 Department of Veterans Affairs Medical Center, Dermatology Service, 1055 Clermont Street, Rm 6A-105, Mail Stop 165, Denver, CO 80220, USA

## Abstract

**Introduction:**

Splinters and spines of plant matter are common foreign bodies in skin wounds of the extremities, and often present embedded in the dermis or subcutaneous tissue. Vegetative foreign bodies are highly inflammatory and, if not completely removed, can cause infection, toxic reactions, or granuloma formation. Older patients are at increased risk for infection from untreated plant foreign bodies. The most common error in plant splinter and spine management is failure to detect their presence.

**Case presentation:**

Here we report a case of cactus spines in an 84-year-old Caucasian man presenting on the right flank as multiple, red papules with spiny extensions. This presentation was unusual both in location and the spinous character of the lesions, and only after punch biopsy analysis was a diagnosis of cactus matter spines made.

**Conclusions:**

Our patient presented with an unusual case of cactus spines that required histopathology for identification. Skin lesions with neglected foreign bodies are a common cause of malpractice claims. If not removed, foreign bodies of the skin, particularly in elderly individuals, can result in inflammatory and infectious sequela. This report underscores the importance of thoroughly evaluating penetrating skin lesions for the presence of foreign bodies, such as splinters and spines.

## Introduction

Plant splinters and spines are penetrating foreign bodies that commonly cause injury to the skin. They may be wood or thorns, and often involve the skin on the extremities. These foreign bodies can release toxins (histamine, acetylcholine or serotonin) or allergens, as well as potentially introduce fungal and bacterial pathogens into the wound [1]. When possible, they should be removed before inflammation or infection occurs. Because plant splinters and spines can penetrate deep into the skin, particularly when entering the skin perpendicularly, they often go undetected [2]. When unrecognized and left unremoved, they can cause inflammation, granuloma formation, and possibly localized or disseminated infection [1,3]. Elderly patients and the immunosuppressed are at increased risk for these negative outcomes [4]. We report a case of an elderly man with an uncharacteristic presentation of asymptomatic and unnoticed vertical and superficial plant spines requiring histopathological review to reach the diagnosis. To our knowledge, this is the first case report describing this unusual presentation for plant matter foreign bodies. This report emphasizes the utility of routine histopathology in plant splinter and spine detection, and underscores the importance of evaluating inexplicable skin wounds for the presence of foreign bodies.

## Case presentation

An 84-year-old Caucasian man presented to the dermatology clinic with a new onset of red bristle-like lesions on the trunk. These lesions were without pain, tenderness, or pruritus. The patient is a diabetic and had a recent history of a painful drug rash secondary to hydrochlorothiazide, which was resolving at the time of examination. Medications used during treatment of his drug rash included predinisone, clobatesol and fluocinonide. In the setting of this resolving, painful drug rash, the new onset bristle-like spines were undetected by our patient, and were instead noted by his wife. Our patient is a retired veteran living with his wife in the rural countryside of the Colorado Front Range region. While he does not garden or directly handle plants, he enjoys the outdoors, and frequently takes walks along the country roads surrounding his home.

**Figure 1:**
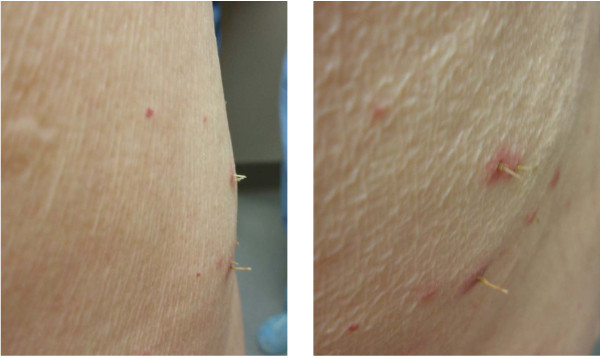
**Erythematous papules on physical examination**. Digital photograph of our patient's right flank demonstrates a cluster of 3 mm erythematous papules. Papules were positive for central ulceration and central spiny growth extensions up to 4 to 5 mm in length. Images captured of lesions on profile (left panel) and close up (right panel).

**Figure 2:**
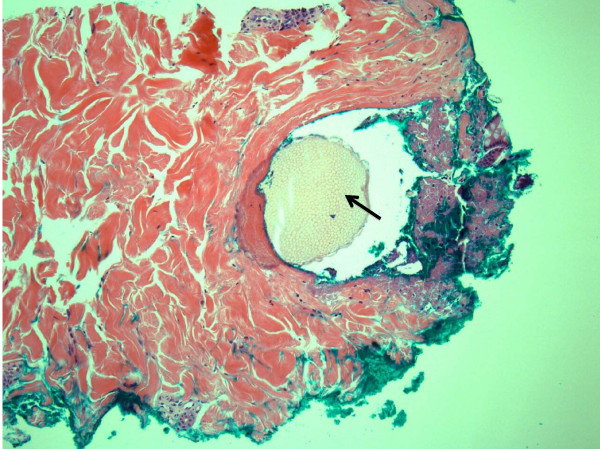
**Punch biopsy of one papule with central foreign body**. Hematoxylin and eosin (H&E)-stained cross-section of one papule. Arrow indicates foreign body. Image captured at 10× magnification.

**Figure 3:**
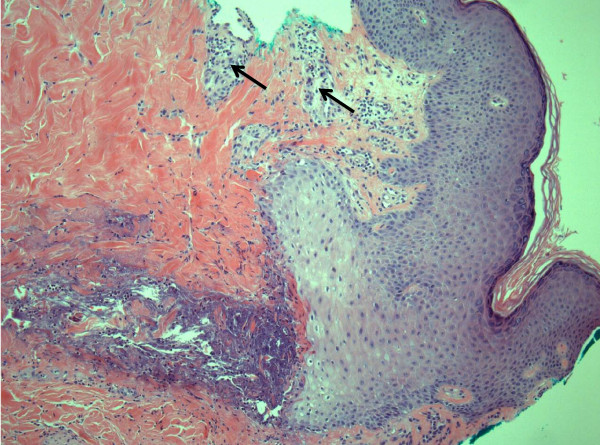
**Punch biopsy of one papule demonstrating inflammatory infiltrate**. H&E-stained cross section of one papule demonstrating infiltrating inflammatory cells (arrow). Image captured at 10× magnification.

**Figure 4:**
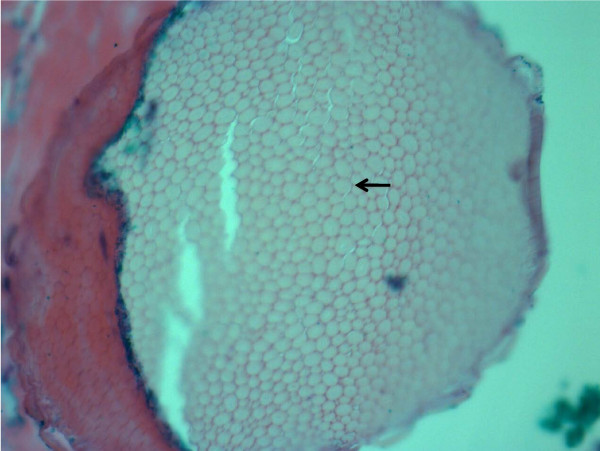
**Microscopic analysis of cactus spine foreign body**. High magnification microscopic examination of the foreign body. Presence of regular repeating hexagonal cell wall structures (arrow) indicates vegetative composition of foreign body consistent with cactus spines [5]. Image captured at 40× magnification.

On examination, 5 to 6 3 mm erythematous papules were grouped along the right flank. Each papule possessed a central thin spine, extending up to 4 to 5 mm in length from the skin (Figure 1). A punch biopsy was performed of a single papule and the differential diagnosis included foreign body, lichen spinulosus or one of the perforating disorders associated with diabetes (i.e. Kyrle disease, perforating folliculitis, reactive perforating collagenosis, and acquired perforating dermatosis). Microscopic analysis revealed the presence of a foreign body (Figure 2) and a surrounding inflammatory infiltrate (Figure 3). The foreign body consisted of a monomorphic and geometric pattern of cell walls, exhibiting the basic morphology of cactus spines [5] (Figure 4). A diagnosis of cactus spines with acute inflammation was thus established.

## Discussion

Foreign body implantation of the skin occurs frequently in both pediatric and adult patient populations. Wood, glass, and metallic splinters are the most common retained foreign bodies, and, if left in the body, can cause inflammation, infection, toxin mediated contact urticaria (stinging nettles) or mechanical irritant dermatitis (glochid dermatitis from cacti such as prickly pears), or granuloma formation [1,2]. Patients may not be aware of the initial injury and at times, deeply penetrating splinters cannot be visualized. In these cases, unfortunately, the only indication of retained foreign material might be swelling, tenderness, a draining sinus or abscess, osteomyleitis, cellulitis, lymphangitis, bursitis, synovitis, or arthritis [6,7].

Factors that influence the outcome of a retained plant matter foreign bodies include the following: length of time since introduced into a wound, the type of foreign body (i.e. wood, glass, or metal), how clean the wound was, and the patient's health status [1]. Acute injury may present with an acute inflammatory infiltrate of neutrophils and eosinophils leading to inflammation and edema, while a granulomatous reaction with foreign-body giant cells might occur in older lesions [8]. Foreign body composition dictates the tissue reaction, as some types of foreign bodies are more toxic and allergenic than others. Organic matter, such as spines and splinters, is highly inflammatory, whereas glass, metal, and plastic are relatively inert materials [6,7]. Foreign body injury can further complicate wounds by introducing bacterial and fungal infection and these should be looked for during histological examination. Risk of infection is increased in the setting of lowered immune status. Notably, elderly and diabetic patients are at increased risk of infection after cutaneous penetrating trauma [4]. Splinters and spines embedded within deeply penetrating wounds can lead to severe infections such as cellulitis, myonecrosis, septic arthritis or osteomyletis [6,7].

To prevent infection or chronic inflammatory reaction, it is recommended that plant matter foreign bodies be removed as soon as possible. The most common error in management of soft tissue foreign bodies is failure to detect their presence [6,9]. While splinters and spines most often present in the superficial or subcutaneous soft tissues of the extremities, larger and deeper splinters can occur and are difficult to detect [3]. In addition to the extent of soft tissue penetration, timing of injury also influences the ability to detect and evaluate splinters. Fresh injuries typically have an injury track that leads to the splinter, facilitating detection and removal. Older injuries, however, may present in the context of infection, inflammation, induration, scarring, or granuloma formation, making foreign body localization difficult [10]. Risk of infection increases with time until a wound is fully healed. Therefore, when possible, wounds should be evaluated for foreign body splinters/spines within 24 hours of injury. Minor, penetrating, skin wounds with neglected foreign bodies are a common cause of malpractice claims [11]. While patients may have a sensation of a foreign body within a wound, they may not be aware of any retained foreign matter [12]. Thus, any wound or lesion that penetrates the skin should be evaluated for the presence of foreign bodies.

This is the first report of superficial and perpendicular cactus matter spines of the flank. Of unique significance, in the setting of a pre-existing drug rash, our patient did not notice the splinters, nor did he recall having come into direct contact with plant matter. The unusual presentation of the splinters required histopathological analysis to arrive at the diagnosis of foreign body splinters of the skin. This report underscores the importance of evaluating wounds and/or lesion that penetrate the skin for the presence of foreign bodies.

## Conclusions

If not completely removed, cactus spines can cause complications such as inflammation, infection, toxin mediated reactions, allergic reactions and granuloma formation. A high index of suspicion is needed in the management of soft tissue foreign bodies as patients often deny history of penetrating injury. Penetrating skin wounds should be evaluated for the presence of foreign bodies, as failure to diagnose and remove splinters can result in patient harm and malpractice.

## Competing interests

The authors declare that they have no competing interests.

## Authors' contributions

AS, SF, LP, and RD analyzed and interpreted the patient data regarding the foreign body of the skin. The manuscript was written by AS and critically evaluated by SF, LP, and RD. All authors read and approved the final manuscript.

## Consent

Written informed consent was obtained from the patient for publication of this case report and any accompanying images. A copy of the written consent is available for review by the Editor-in-Chief of this journal.
